# Long-term performance and microbial insights of an iron–carbon enhanced CW-MFC and shallow sand filtration system for rural greywater treatment

**DOI:** 10.1039/d5ra09271j

**Published:** 2026-01-30

**Authors:** Zhiwei Zhu, Song Chen, Yahui Li, Junyong He, Yulian Li, Peidong Hong, Chao Xie, Zijian Wu, Jiandong Lu, Dandan Yang, Lingtao Kong

**Affiliations:** a University of Science and Technology of China Hefei 230026 PR China; b Environmental Materials and Pollution Control Laboratory, Institute of Solid State Physics, HFIPS, Chinese Academy of Sciences Hefei 230031 PR China liyh@issp.ac.cn ltkong@iim.ac.cn +86-551-65591415; c Key Laboratory of Water Pollution Control and Wastewater Reuse of Anhui Province, Anhui Provincial Key Laboratory of Environmental Pollution Control and Resource Reuse, Anhui Jianzhu University Hefei 230601 China; d Xi'an University of Architecture and Technology Xi'an 710055 China

## Abstract

A pilot-scale integrated system combining an iron–carbon-enhanced anode constructed wetland-microbial fuel cell with shallow sand filtration (ICCW/MFC-SSF) was developed for decentralized rural greywater treatment. During an eight-month field trial, the system demonstrated robust and stable performance. It achieved high removal efficiencies for COD (92.3%), TN (82.6%), NH_4_^+^-N (97.1%), SS (88.1%), turbidity (97.9%), and fecal coliforms (97.8%), with effluent meeting Chinese irrigation standards. The iron–carbon anode may have enhanced electron transfer and oxidative degradation, while the SS may have contributed to further nitrogen removal *via* reaeration. Dissolved organic matter (DOM) analysis revealed a shift from protein-like to refractory fulvic-like substances. Microbial community analysis indicated niche differentiation, with Proteobacteria dominant in the cathode and Firmicutes enriched in the anode. However, phosphorus removal declined over time due to adsorption saturation, highlighting a key limitation for long-term application.

## Abstract 2

A pilot-scale iron–carbon enhanced constructed wetland-microbial fuel cell coupled with shallow sand filtration demonstrated stable, efficient removal of organics, nutrients and pathogens from rural greywater during long-term field operation.

## Graphical abstract

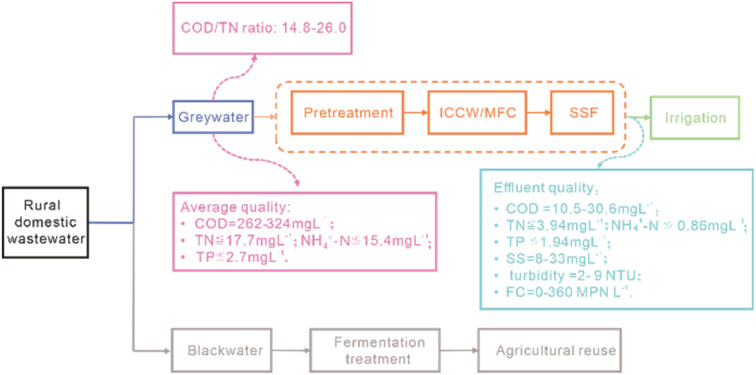

## 1. Introduction

With the improvement of living standards and changes in lifestyle in rural areas, the discharge of domestic greywater (from washing, laundry, kitchen, *etc.*) has been increasing annually.^1–3^ This type of decentralized wastewater is characterized by high flow fluctuation, elevated organic loads, and complex nutrient composition. Direct discharge can lead to eutrophication of water bodies and pathogen transmission, posing risks to both public health and the aquatic environment.^4,5^ Furthermore, traditional combined collection and centralized treatment systems face challenges such as high pipeline costs, low resource recovery, and significant energy consumption.^6–8^ In this context, source separation sanitation systems offer a sustainable solution to these challenges: blackwater can be recycled as agricultural fertilizer after proper treatment, while greywater can be treated on-site and reused, substantially reducing freshwater consumption.^9–11^

Constructed wetland-microbial fuel cells (CW-MFCs) represent an emerging ecological technology that integrates the pollutant removal capabilities of wetlands with the electricity generation function of microbial fuel cells.^12–14^ This integrated system offers advantages such as low construction costs, simple operation and maintenance, and no secondary pollution, making it particularly suitable for rural areas with limited infrastructure. However, conventional CW-MFCs encounter several technical bottlenecks in practical applications. These include limited mass transfer efficiency, susceptibility of nitrogen and phosphorus removal to environmental factors, and inadequate adaptability to complex wastewater compositions.^15–17^

In recent years, iron–carbon micro-electrolysis enhancement and shallow sand filtration (SSF) have been introduced to improve CW-MFC performance. Iron–carbon materials generate reactive species (*e.g.*, Fe^2+^/Fe^3+^, ˙OH) through micro-electrolysis, enhancing pollutant oxidation, coagulation, and electron transfer efficiency.^18,19^ Meanwhile, SSF further optimizes effluent quality, particularly nitrogen transformation, through atmospheric reoxygenation and biofilm processes. Nevertheless, most existing studies are confined to laboratory-scale experiments using synthetic wastewater. They also lack systematic understanding of the long-term transformation mechanisms of pollutants, the evolution of dissolved organic matter (DOM), and the response of microbial communities in real greywater treatment systems.

To address these research gaps, this study innovatively established a pilot-scale iron–carbon-enhanced anode CW-MFC coupled with a shallow sand filter (ICCW/MFC-SSF) and conducted an eight-month field experiment. The objectives were to: (1) systematically evaluate the long-term treatment performance of the system for rural greywater under real-world conditions, with a primary focus on water quality remediation rather than bioenergy recovery; (2) elucidate the transformation characteristics of DOM fractions during the treatment process; and (3) investigate the response mechanisms of microbial community structure to process enhancement. The MFC configuration was employed primarily as an integrated strategy to create redox zones and electrode habitats for enhancing microbial processes and pollutant removal, consistent with its application in prior hybrid wetland systems. Quantitative evaluation of bioelectrical output, while a characteristic of MFCs, was not the central aim of this field deployment but has been demonstrated for the iron–carbon anode in controlled laboratory studies (see companion article^20^). This research aims to provide an efficient and stable technical solution, along with theoretical support, for decentralized rural greywater treatment. It should be noted that while specific electrochemical mechanisms (*e.g.*, micro-electrolysis, *in situ* radical generation) are discussed based on design principles and supporting laboratory evidence (*e.g.*, our companion study^20^), the primary focus of this field trial is on evaluating integrated system performance, DOM fate, and microbial ecology under long-term, real-world conditions.

## 2. Materials and methods

### 2.1. Process design and operation

**Fig. 1:**
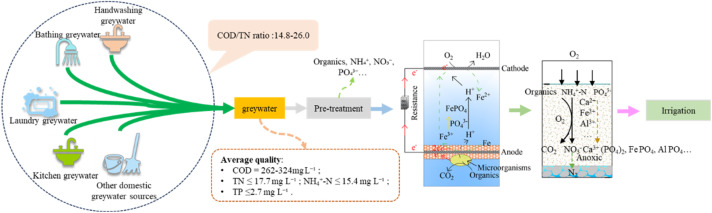
Flowchart of domestic greywater treatment and resource recovery.

This pilot-scale study was conducted under real-field conditions at a single household in a natural village of Hefei City, Anhui Province, China, to evaluate an integrated greywater treatment system. The household, with 3–4 residents, was equipped with a source-separation-based eco-sanitation system where domestic greywater was collected and treated on-site. As illustrated in Fig. 1, the treatment train consisted of an equalization tank, a CW-MFC, and a SSF unit, with the final effluent being discharged for agricultural irrigation. An equalization tank was installed as the primary unit to buffer the significant temporal variations in hydraulic and pollutant loads typical of decentralized greywater sources.

The CW-MFC, with dimensions of 1000 mm × 500 mm × 1500 mm (*L* × *W* × *H*) and an effective bed height of 1200 mm, featured a layered configuration. This included a 100 mm gravel support layer (10–20 mm), an 800 mm main gravel layer (5–10 mm), and a 300 mm planting layer. A critical design feature was the placement of the anode, which was encased within a dedicated 100 mm layer of iron–carbon filler (5–8 mm); both anode and cathode were fabricated from stainless-steel-mesh-wrapped carbon felt and connected with an external 2000 Ω resistor. The subsequent SSF unit (500 mm × 500 mm × 700 mm), with a 300 mm effective depth of river sand (<1 mm), received the CW-MFC effluent by gravity for further polishing. Parallel factor analysis (PARAFAC) of DOM was performed in MATLAB R2018b.

The system operated under natural inflow conditions with a HRT of approximately 3 days in the CW-MFC; the HRT in the SSF was negligible due to its rapid drainage. Common reed (*Phragmites australis*) was planted in both the CW-MFC and the SSF unit at a density of 20 plants per m^2^. The experimental monitoring period reported herein spanned from May to December 2024, with water samples collected at biweekly intervals for comprehensive analysis. During this period, the ambient temperature ranged from −5 °C in winter to 40 °C in summer. The daily greywater flow rate varied from 12 to 204 L d^−1^, reflecting the typical fluctuation in water usage of a rural household.

### 2.2. Analytical methods

#### 2.2.1. Analytical parameters

COD was determined using the rapid sealed digestion method. TN was measured *via* alkaline potassium persulfate digestion followed by spectrophotometric detection. NH_4_^+^-N was analyzed by Nessler's reagent spectrophotometry, NO_3_^−^-N by ultraviolet spectrophotometry, and NO_2_^−^-N by diazotization-coupling spectrophotometry. TP was quantified using the molybdate spectrophotometric method, while SS were measured gravimetrically. pH values were recorded with a Hach HQ30d water quality analyzer. Fecal coliforms were enumerated *via* a rapid test kit (Guangdong Da Yuan Oasis Food Safety Technology Co., Ltd, China). 3D-EEM fluorescence spectroscopy was performed using a Hitachi F7000 spectrofluorometer (Japan) equipped with a xenon lamp. The photomultiplier tube voltage was set at 500 V, with both excitation and emission slit widths fixed at 5 nm. Scanning ranges were 220–400 nm for excitation and 280–500 nm for emission, with 5 nm intervals and a scan speed of 2400 nm min^−1^.

NH_4_^+^-N, NO_3_^−^-N and NO_2_^−^-N were determined using potassium chloride extraction followed by flow injection analysis (FIA). Soil pH was measured by the potentiometric method. Organic matter content was analyzed *via* the sulfuric acid–potassium dichromate oxidation method. TN was quantified using the Kjeldahl method, while TP was measured after sulfuric acid–perchloric acid digestion followed by molybdenum-antimony anti-colorimetric determination.

#### 2.2.2. Microbial community analysis

The biofilm samples collected from the filler surfaces were processed by a third-party specialized laboratory for DNA extraction and quality control. Subsequent high-throughput sequencing of the hypervariable regions was performed using the Illumina HiSeq platform (based on 16S rRNA gene sequencing). The obtained sequences were analyzed to determine microbial diversity, as well as taxonomic composition and relative abundance within the communities.

#### 2.2.3. Statistical analysis

All water quality data were based on 16 independent sampling events conducted at biweekly intervals over the eight-month monitoring period. Descriptive statistics (mean ± standard deviation) were used to summarize pollutant concentrations and removal efficiencies across different treatment units (Fig. 3). To assess the significance of differences among treatment stages (influent, CW-MFC effluent, and SSF effluent), one-way analysis of variance (ANOVA) was performed for each pollutant parameter (COD, TN, NH_4_^+^-N, TP). Where ANOVA indicated a significant overall difference (*p* < 0.05), post-hoc pairwise comparisons were conducted using Tukey's Honestly Significant Difference (HSD) test. The results of these comparisons are indicated by different lowercase letters (a, b, c) in Fig. 3. All statistical tests and graphical representations were performed using OriginPro 2025 (OriginLab Corporation, USA).

For the correlation analysis presented in Fig. 4, Pearson's correlation coefficients were calculated based on the same 16 repeated measurements for each water quality parameter. Normality of the data distribution was assessed using the Shapiro–Wilk test prior to correlation analysis. The assumption of homoscedasticity is not required for Pearson's correlation, which examines linear relationships between continuous variables without grouping or mean comparison.

## 3. Results and discussion

### 3.1. Contaminant removal performance

#### 3.1.1. COD

The integrated iron–carbon anode CW-MFC and SSF system achieved exceptional COD removal efficiency (90.1–94.8%) for rural greywater treatment, effectively reducing COD concentrations from 261.9–323.6 mg L^−1^ (characteristic of such wastewater and consistent with literature values^21^) to 10.5–30.6 mg L^−1^. The findings suggest that, as the primary treatment unit, the CW-MFC contributed substantially to total COD removal. While the specific quantitative contributions of electrochemical oxidation, microbial degradation, and physical adsorption cannot be decoupled without controlled comparisons (*e.g.*, open-circuit operation, non-iron–carbon fillers), the high removal within this unit likely results from potential synergistic mechanisms. These may include (i) electrochemical oxidation driven by the anode potential, (ii) chemical oxidation *via* reactive oxygen species (*e.g.*, hydroxyl radicals, ˙OH) generated through iron redox cycling (Fe^0^ → Fe^2+^; Fe^2+^ + H_2_O_2_ → Fe^3+^ + ˙OH + OH^−^ in the presence of *in situ* produced or ambient oxidants) at the iron–carbon filler, microbial degradation by anode-enriched biofilms (predominantly Proteobacteria and Bacteroidetes),^22,23^ and physical adsorption within the porous iron–carbon filler. The iron–carbon micro-electrolysis core likely facilitates a continuous supply of Fe^2+^, which can act as an electron donor in the MFC anode chamber while also participating in Fenton-like oxidative pathways. The subsequent SSF unit may enhance treatment performance by providing an additional 10% removal through colloidal organic matter interception by fine sand and secondary biodegradation *via* attached biofilms.^24^ Collectively, this demonstrates a robust synergistic system capable of maintaining stable operation under influent fluctuations.

#### 3.1.2. Nitrogen

**Fig. 2:**
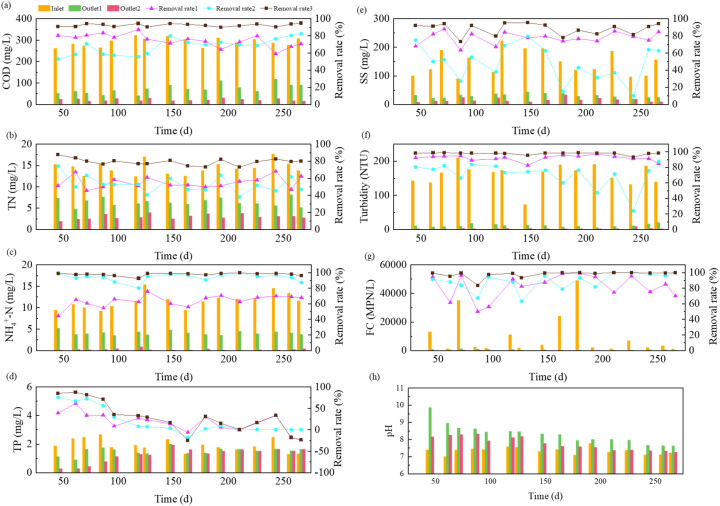
Pollutant removal performance in systems: (a) COD, (b) TN, (c) NH_4_^+^-N, (d) TP, (e) SS, (f) turbidity, (g) FC, (h) pH.

As shown in Fig. 2b and c, the coupled system exhibited excellent nitrogen removal performance, reducing TN and NH_4_^+^-N from 12.46–17.66 mg L^−1^ and 9.24–15.38 mg L^−1^ (74.2–87.1% of TN) to 2.51–3.94 mg L^−1^ and 0.01–0.86 mg L^−1^, respectively. This corresponds to average removal efficiencies of 79.3% for TN and 97.8% for NH_4_^+^-N. In the Fe–C enhanced anode CW-MFC unit, nitrogen removal may be achieved through the following potential synergistic mechanisms: physicochemical adsorption by hierarchical porous fillers,^25^ chemo-electrochemically promoted nitrification and denitrification driven by a 0.3–0.5 V micro-electric field and possibly enhanced by iron-mediated redox shuttling (*e.g.*, Fe^3+^/Fe^2+^ couples facilitating electron transfer for anaerobic ammonium oxidation or denitrification),^26^ and plant uptake.^27^ The subsequent shallow sand filtration may have enhanced nitrification (>95% residual NH_4_^+^-N conversion), which could be attributed to efficient reaeration.^28^ Notably, sustained TN removal (>70%) confirmed that the process relied predominantly on biological transformation rather than adsorption. This electrochemically-enhanced nitrogen removal strategy offers a novel pathway for decentralized wastewater treatment. In this study, the term “electrochemical enhancement” specifically refers to the incorporation of electrode-associated processes and redox gradients into the wetland matrix to stimulate microbial activity—as reflected in shifts in microbial community structure—rather than the harvesting of electrical energy. It should also be noted that within this integrated system, the contributions of the micro-electric field, iron–carbon reactions, and conventional wetland processes to nitrogen transformation are intricately interwoven and challenging to decouple quantitatively. Therefore, future studies employing controlled experimental designs are warranted to dissect the individual roles and synergistic effects of these mechanisms.

#### 3.1.3. TP

As shown in Fig. 2d, the influent TP concentration varied between 1.29 and 2.66 mg L^−1^, with removal efficiency exhibiting three distinct phases. During the initial phase (0–70 days), high TP removal (87.54% on average) may be achieved through chemical precipitation as iron–phosphate complexes (*e.g.*, FePO_4_, Fe_3_(PO_4_)_2_) *via* Fe^2+^/Fe^3+^ ions released from iron–carbon micro-electrolysis,^29^ physical adsorption by filler media,^30^ and plant uptake.^31^ However, over prolonged operation (70–180 days), TP removal declined progressively to negligible levels. This decline is attributable to adsorption site saturation,^32^ depletion of reactive zero-valent iron (Fe^0^) and soluble Fe^2+^ in the iron–carbon materials leading to cessation of precipitation,^33^ and cessation of plant uptake under low winter temperatures.^34^ This phenomenon aligns with the findings of Ho *et al.*, who reported that natural zeolite adsorption capacity reached saturation when the cumulative liquid volume per unit weight exceeded 0.2 L g^−1^ in a post-treatment system for MFCs.^35^ These dynamics underscore the inherent limitation of ecologically coupled phosphorus removal, which relies heavily on finite adsorption capacity and seasonal plant activity.

#### 3.1.4. SS and turbidity

As shown in Fig. 2e and f, the coupled system effectively reduced SS and turbidity from 90–223 mg L^−1^ and 74–210 NTU to average effluent concentrations of 16 mg L^−1^ and 3 NTU, achieving removal efficiencies of 88.10% and 97.90%, respectively. The Fe–C enhanced anode CW-MFC unit may have contributed over 80% of the removal efficiency through the following three potential synergistic mechanisms: electrocoagulation *via* Fe^2+^/Fe^3+^-induced charge neutralization,^36^ bioflocculation enhanced by anode biofilm-derived extracellular polymeric substances,^37^ and progressive particle retention by multi-layer filtration media.^38^ The subsequent shallow sand filter provided precision filtration for the remaining 20%. Together, they established a robust “coarse-to-fine” hierarchical removal pattern that ensured consistently high SS and turbidity elimination.

#### 3.1.5. Fecal coliforms

The integrated system demonstrated remarkable fecal coliform removal, reducing concentrations from 1100–49 000 MPN L^−1^ to 0–360 MPN L^−1^ with 97.82% average efficiency. The Fe–C enhanced anode CW-MFC may be contribute 82.10% of this removal through multiple potential inactivation pathways: electrochemical disinfection *via* membrane disruption under a microelectric field potentially generated in the system,^39^ reactive oxygen species (*e.g.*, ˙OH) oxidation,^40^ competitive inhibition by antimicrobial-producing biofilms,^41^ and physical retention by filtration media. The subsequent SSF further eliminated residual pathogens (4.78% contribution) through biofilm competition and fine-filtration. Thus, the system established a reliable multi-barrier strategy for safe greywater reuse.

#### 3.1.6. pH

As shown in Fig. 2h, the influent pH remained neutral to weakly alkaline (7.09–7.77). During initial operation (0–90 days), effluent pH from the Fe–C enhanced CW-MFC exceeded 8.5 due to OH^−^ generation from cathode reactions and iron corrosion. With prolonged operation (90–180 days), microbial acid production progressively neutralized alkalinity, stabilizing pH at 7.6–8.2. The subsequent SSF effluent maintained a consistently lower pH (7.2–8.0, *Δ* = 0.2–0.4 units), which may be attributed to proton release from aerobic degradation,^42^ CO_2_-derived carbonic acid formation,^43^ and nitrification processes.^44^ Overall, this systematic pH evolution demonstrates the establishment of dynamic biogeochemical equilibrium across treatment stages.

### 3.2. Segmental removal characteristics and synergistic mechanisms of coupled processes

#### 3.2.1. Removal performance and statistical significance by segment

**Fig. 3:**
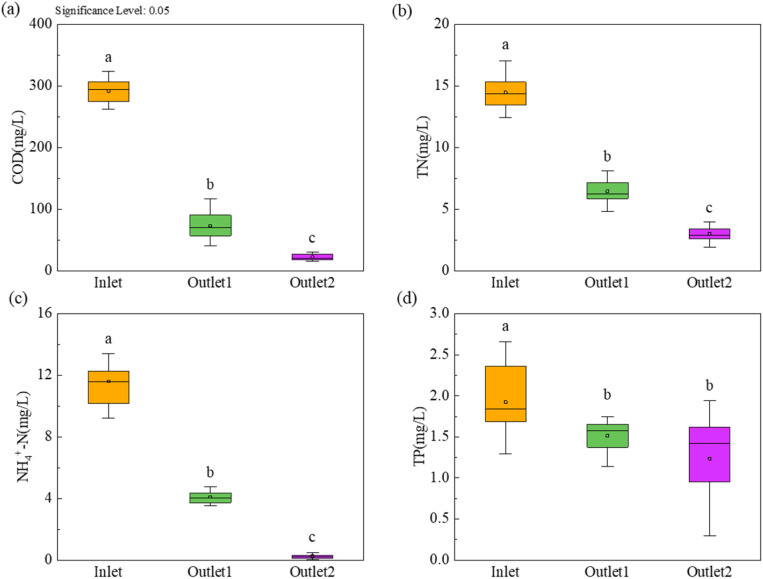
The average removal performance and statistical analysis of major pollutants in different processing units: (a) COD, (b) TN, (c) NH_4_^+^-N, and (d) TP [means with different letters (a, b, c) differ significantly (*p* < 0.05)].

As shown in Fig. 3, stage-wise comparison of pollutant levels revealed distinct removal patterns across the treatment train. Significant reductions (*p* < 0.05) in COD, TN, NH_4_^+^-N, and TP between influent and CW-MFC effluent underscored the primary treatment role of the CW-MFC unit. Further significant decreases in COD, TN, and NH_4_^+^-N between CW-MFC and SSF effluents highlighted the polishing capacity of the sand filter, whereas TP removal showed no significant improvement. This differential behavior suggests that the SSF unit may enhance the removal of organic matter and nitrogen through reaeration and biofilm activity. However, its limited contribution to phosphorus elimination is likely attributable to the absence of metal ions for phosphate precipitation,^45^ unfavorable aerobic conditions for polyphosphate-accumulating organisms (PAOs),^46^ and insufficient hydraulic retention time for complete chemical precipitation.^47^

#### 3.2.2. Principal component and correlation analysis

**Fig. 4:**
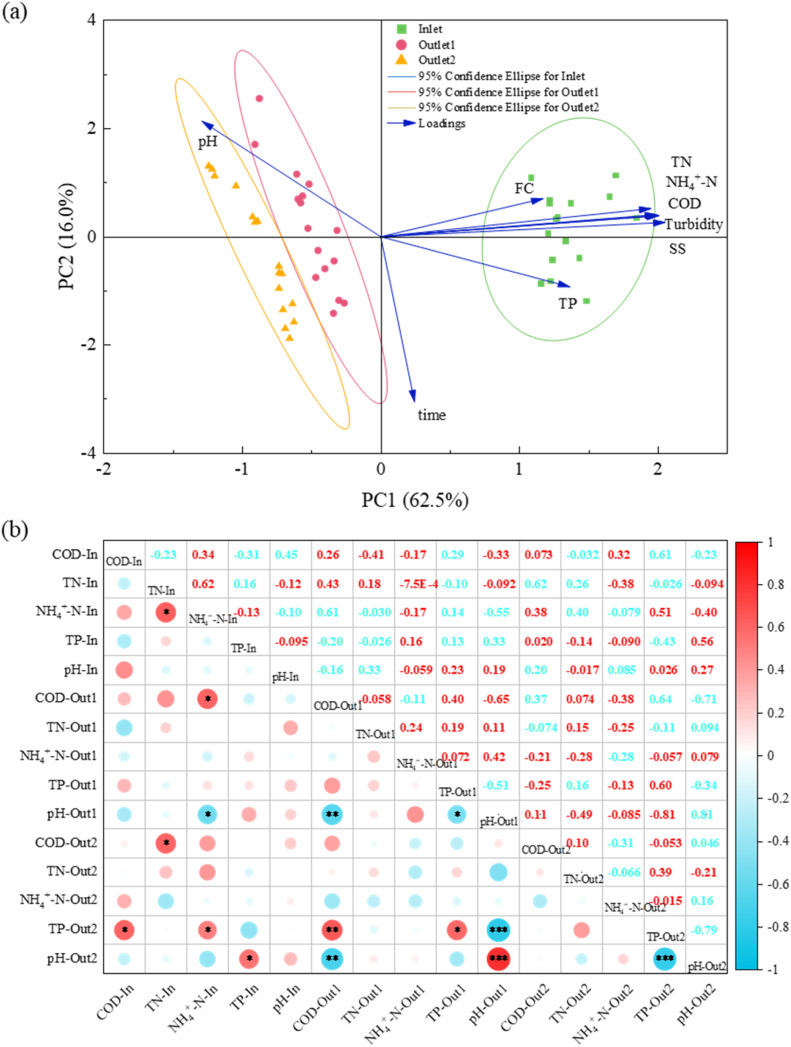
(a) PCA and (b) Pearson's correlation analysis of different water quality parameters across system stages (*p ≤ 0.05, **p ≤ 0.01, ***p ≤ 0.001, n.s. = no significance).

Principal component and correlation analyses (Fig. 4) revealed systematic water quality evolution across treatment stages. PCA clearly separated influent, CW-MFC effluent, and SSF effluent along PC1 (62.5% variance). Here, the pronounced segregation between influent and CW-MFC effluent underscoring the latter's pivotal role in pollutant transformation. Key parameters (COD, TN, NH_4_^+^-N) strongly correlated with PC1 and decreased progressively along the treatment train, confirming continuous purification. Correlation analysis further identified: (1) ammonium-dominated nitrogen composition (TN-NH_4_^+^-N: *r* = 0.62, *p* < 0.05); (2) acid generation during organic degradation (pH-COD: *r* = −0.65, *p* < 0.01); and (3) alkaline-enhanced phosphate precipitation (TP-pH: *r* = −0.79, *p* < 0.001).^48^ Competitive carbon–nitrogen removal was evidenced by negative COD-nitrogen removal correlations, positive residual nitrogen-COD relationships, and declining COD/TN ratios. Additionally, system-wide pH consistency (*r* = 0.81, *p* < 0.001) and strengthened TP-pH correlation collectively governed pollutant fate. These results elucidate the synergistic role of electrochemically enhanced oxidation, biodegradation, and chemical precipitation in the integrated system.

### 3.3. DOM transformation characteristics

**Fig. 5:**
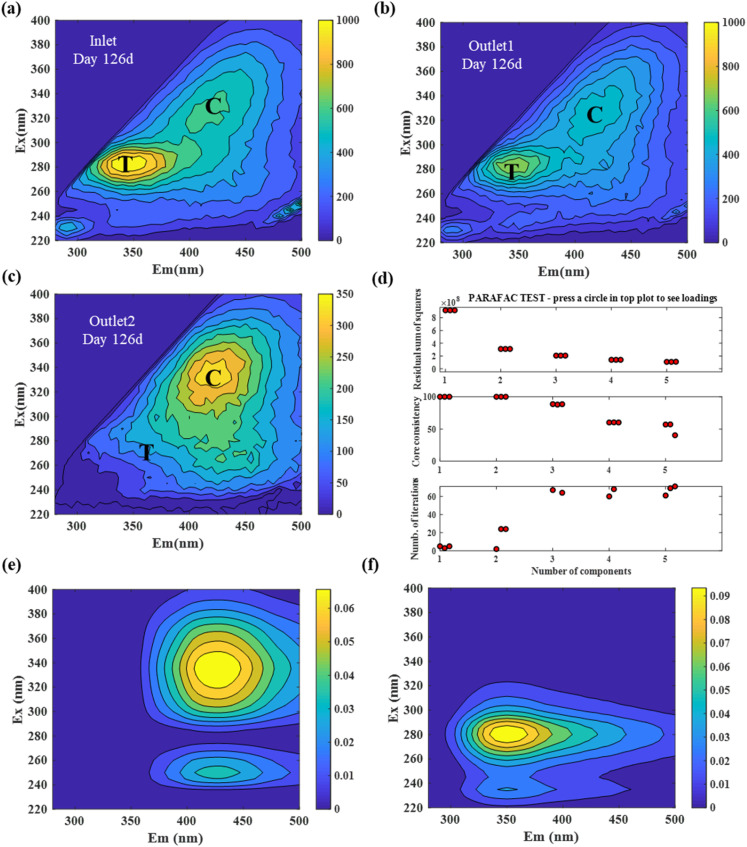
(a–c) 3D-EEM contours of (a) inlet, (b) CW-MFC outlet, and (c) SSF outlet; (d) PARAFAC model residuals and core consistency test; (e and f) resolved fluorescent components: (e) humic/fulvic-like and (f) protein/tryptophan-like signatures. The identified peaks correspond to characteristic DOM fractions: peak T (Ex/Em = 280/350 nm) represents biodegradable protein-like substances, while peak C (Ex/Em = 330/430 nm) indicates recalcitrant fulvic-like components. The spatial shift from protein-like dominance in the influent to fulvic-like prevalence in the final effluent illustrates the effective degradation of labile organics and the accumulation of stable, refractory fractions along the treatment train.

Spatiotemporal evolution of DOM was tracked using 3D-EEM fluorescence spectroscopy coupled with PARAFAC modeling during system operation (Fig. 5a–f). Two characteristic peaks were identified: peak T (Ex/Em = 280/350 nm), indicative of protein-like/tryptophan-like substances, and peak C (Ex/Em = 330/430 nm), characteristic of fulvic-like components. While both influent and CW-MFC effluent were dominated by protein-like signals—reflecting active microbial metabolism and generation of proteinaceous metabolites^49^—the SSF effluent exhibited a distinct compositional shift toward fulvic-like dominance. This transition suggests the accumulation of recalcitrant DOM with aliphatic and minor aromatic constituents, typically derived from incomplete biodegradation or from the chemical transformation of labile organics. The iron-enhanced environment in the CW-MFC may promote oxidative cleavage of proteinaceous and other high molecular weight compounds, leading to partial oxidation and the formation of more refractory, fulvic-like structures that are less susceptible to further biological degradation.^50^

PARAFAC modeling validated two major fluorescent components: C1 (fulvic-like) and C2 (protein-like). The progressive shift from biodegradable protein-like materials to refractory fulvic-like substances along the treatment train aligns with established organic transformation pathways in biological treatment systems.^51^ This shift likely results from a combination of biological consumption of labile fractions and iron-mediated chemical oxidation/coagulation that selectively removes or transforms certain DOM components, leaving behind a more oxidized, condensed fulvic-like pool. These findings underscore the persistence of fulvic-like fractions in the final effluent, providing molecular-level insights for optimizing organic removal performance in advanced treatment systems.

Overall, the shift from biodegradable protein-like to refractory fulvic-like DOM highlights the effective degradation of labile organics in the system, while indicating the accumulation of stable, recalcitrant fractions that may require further attention in long-term operation.

### 3.4. Spatial distribution patterns of nutrients in SSF

**Fig. 6:**
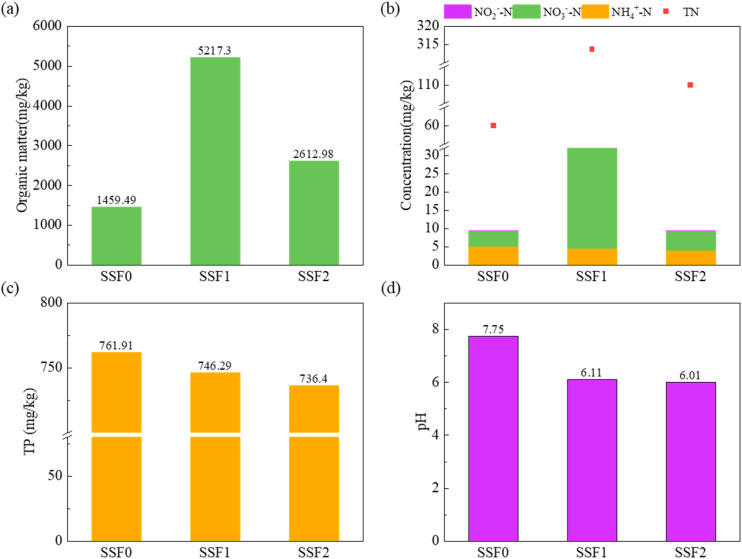
Variations of nutrient concentrations in SSF system: (a) COD, (b) nitrogen species (TN, NH_4_^+^-N, NO_2_^−^-N, NO_3_^−^-N), (c) TP, (d) pH. The vertical profiles reflect redox-stratified removal mechanisms: surface layers show high organic interception and chemical phosphorus precipitation, mid-depth zones exhibit aerobic nitrification (evidenced by NH_4_^+^-N decrease and NO_3_^−^-N peak), and deeper anoxic layers favor fermentation and phosphorus retention under acidic conditions. These patterns demonstrate the SSF's role as a multi-functional polishing unit through spatially structured biogeochemical processes.

The SSF served as a vital polishing unit, achieving advanced removal of residual pollutants from the Fe–C enhanced CW-MFC effluent. Spatial profiling revealed distinct stratification of organic matter (Fig. 6a), with content increasing from 1459.5 mg kg^−1^ at the surface to a peak of 5217.3 mg kg^−1^ at 100 mm depth, then declining to 2613.0 mg kg^−1^ at 200 mm. This distribution reflects initial interception of macromolecules at the surface, enhanced capture and degradation by mature biofilms in the middle layer, and reduced microbial activity under anoxic conditions at greater depths.

Nitrogen transformation exhibited strong redox-dependent behavior (Fig. 6b). NH_4_^+^-N decreased steadily with depth, indicating aerobic nitrification, while NO_3_^−^-N showed a unimodal distribution peaking in the oxygen-rich middle layer. The consistently low NO_2_^−^-N (<0.02 mg kg^−1^) across all depths confirms efficient nitrite oxidation by nitrite-oxidizing bacteria.^52^ Phosphorus was primarily retained in surface layers (761.9 → 736.4 mg kg^−1^) *via* chemical precipitation with Ca^2+^/Mg^2+^, forming stable Ca_5_(PO_4_)_3_OH-like complexes.^53^

Vertical pH variation supported these mechanisms (Fig. 6d): surface alkalinity (pH ≈ 7.75) reflected CW-MFC effluent characteristics, while acidification in deeper layers (pH ≈ 6.01–6.11) resulted from anaerobic fermentation.^54^ This structured chemo-biological zonation enables multi-functional purification: chemical phosphorus immobilization near the surface, aerobic nitrification in mid-depth biofilms, and phosphorus retention under acidic conditions at depth. Consequently, these insights support optimized SSF design through controlled aerobic zone depth (100–150 mm) and surface-amended Ca-rich media to enhance synergistic pollutant removal.

### 3.5. Microbial community structure and function

#### 3.5.1. Microbial community differentiation

Alpha diversity analysis revealed distinct spatial stratification of microbial communities in the ICCW/MFC-SSF system (Table 1). High-throughput sequencing showed excellent coverage (>0.99) across all samples. Within the CW-MFC, the cathode supported significantly greater richness (ACE: 1427; Chao1: 1377) and diversity (Shannon: 7.64) than the anode (ACE: 945; Chao1: 958; Shannon: 5.91). This reflects its electron-accepting niche that sustains diverse aerobic, facultative anaerobic, and electroactive bacteria.^55^ The SSF unit exhibited a characteristic U-shaped diversity profile along the depth gradient. The middle layer (100 mm) showed reduced richness (ACE = 693, Chao1 = 704) and diversity (Shannon = 8.02) compared to the surface (ACE = 2299, Chao1 = 2298, Shannon = 9.25), while the deep layer (200 mm) regained higher microbial complexity (ACE = 3504, Chao1 = 3493, Shannon = 9.82). This pattern corresponds to redox zonation: oxygen limitation in the middle layer restricted obligate aerobes, while anoxic conditions at depth promoted anaerobic specialists.^56^ Consistently high Simpson indices (0.98–0.99) indicated stable community evenness, suggesting well-established niche partitioning during long-term operation.^57^ In summary, these findings highlight how electrochemical enhancement and redox stratification collectively shape microbial community structure, providing crucial insights for optimizing biofiltration performance. The distinct communities observed, particularly the enrichment of Firmicutes at the anode and Proteobacteria at the cathode, are consistent with purported bioelectrochemical and redox niches. However, confirming that these shifts are directly driven by the MFC operation rather than by inherent anode/cathode environmental differences would require controlled experiments (*e.g.*, open-circuit controls).

**Table 1:** Results of alpha diversity indices

Sample ID	ACE	Chao1	Shannon	Simpson	Coverage
SSF0	2299	2298	9.25	0.99	1.00
SSF1	693	704	8.02	0.98	1.00
SSF2	3504	3493	9.82	0.99	0.99
Cathode	1427	1377	7.64	0.95	1.00
Anode	945	958	5.91	0.98	1.00

**Fig. 7:**
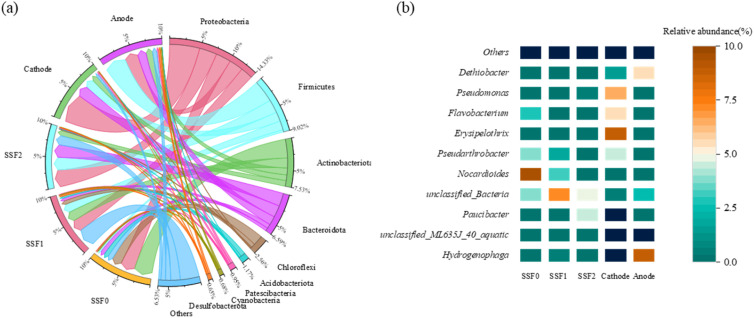
Winter microbial community composition (a) at the phylum level and (b) at the genus level.

Phylum-level analysis (Fig. 7a) revealed Proteobacteria as the dominant taxon across all samples (22.50–48.41%), reflecting its metabolic versatility. Distinct niche differentiation was observed between electrodes: Proteobacteria predominated at the cathode (48.41%), potentially favored by its electron-accepting role, whereas Firmicutes dominated the anode (35.40%), consistent with their known function in nitrogen removal under anoxic conditions and their reported enrichment in Fe–C enhanced electrochemical systems.^19,58^ The distinct enrichment of Firmicutes at the anode may be linked not only to anoxic conditions but also to the availability of iron species (Fe^2+^/Fe^3+^) that can serve as alternative electron acceptors or donors, potentially supporting iron-reducing or iron-oxidizing metabolites that contribute to nitrogen cycling. The enrichment of Proteobacteria in the cathode region of our study, which is associated with electrochemical driving, is consistent with the observations of Sanaei *et al.*, who reported an increase in microbial diversity and abundance following electrode insertion.^59^ Bacteroidota remained at low abundance (4.85–20.98%), aligning with limited phosphorus removal, as this phylum includes phosphate-accumulating organisms.^60^ In the SSF, Chloroflexi decreased significantly with depth (16.32% to 1.54%), likely influenced by organic carbon availability—favoring autotrophic growth in shallower layers.^61^ At the genus level (Fig. 7b), temperature-induced functional specialization was evident. The cathode was enriched with *Hydrogenophaga* (14.73%), *Paucibacter* (14.93%), and unclassified ML635J-40-aquatic_group (12.08%), all involved in electroactivity and organics degradation.^62,63^ The anode also hosted these taxa but at lower abundances, except for *Dethiobacter* (5.64%), indicating redox-specific adaptation. Strikingly, the unclassified group accounted for 23.61% of CW-MFC communities but was negligible in the SSF, highlighting system-specific functional segregation. SSF communities were dominated by *Nocardioides* and *Pseudarthrobacter*, both showing depth-dependent declines and known for degrading refractory organics and detoxifying pollutants.^64^ Taken together, these results demonstrate how redox zonation, carbon gradients, and operational temperature collectively shape microbial structure and function, underpinning pollutant removal mechanisms in integrated bioelectrochemical-filtration systems.

In summary, the distinct microbial differentiation between electrodes and along the SSF depth reflects a functional adaptation to local redox conditions, which supports the system's ability to simultaneously remove carbon, nitrogen, and other pollutants through partitioned metabolic pathways.

#### 3.5.2. Functional prediction

**Fig. 8:**
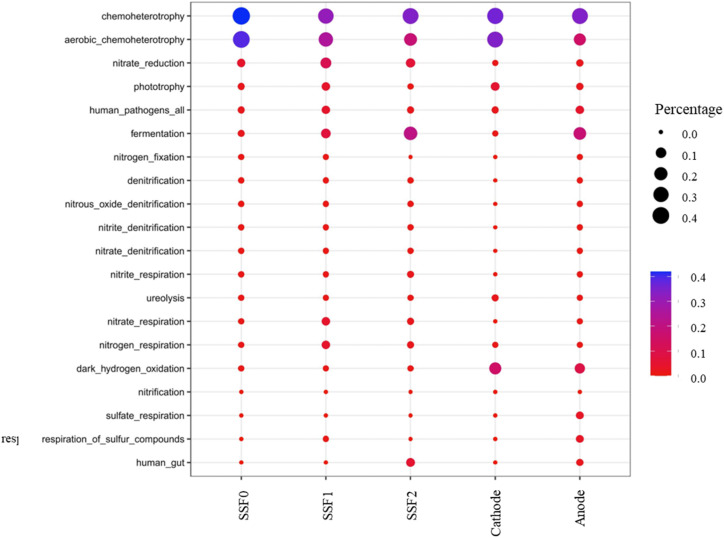
Predicted ecological functions based on FAPROTAX.

FAPROTAX analysis revealed systematic metabolic partitioning across the integrated system (Fig. 8). Core functions included chemoheterotrophy (25.7–32.1%), aerobic chemoheterotrophy (13.5–30.4%), fermentation (1.1–16.1%), and dark hydrogen oxidation (6.75–12.76%). Cathodes favored aerobic processes (aerobic chemoheterotrophy: 28.70%; dark hydrogen oxidation: 12.76%), while anodes exhibited enhanced fermentation (13.81%), reflecting redox-driven niche specialization.^65^

The SSF displayed vertical metabolic stratification: aerobic chemoheterotrophy decreased from 30.4% to 13.5% with depth, while fermentation increased from 1.8% to 16.1%, demonstrating redox-gradient selection. Chemoheterotrophy showed a V-shaped distribution (32.1% → 21.0% → 25.7%), potentially influenced by surface-derived phototrophic products and deep-layer fermentation.^66^ Notably, nitrate reduction was 5.9-fold higher in the SSF (14.8%) than in the CW-MFC (2.5%), confirming its dominant denitrification role.

These results highlight how electrochemically driven aerobic processes in cathodes and anaerobic reduction in SSF can be strategically coupled to enhance concurrent carbon and nitrogen removal in constructed wetland systems. Taken together, the predicted functional profiles reinforce that the integration of electrochemical enhancement and redox stratification promotes complementary microbial metabolisms, thereby improving the overall treatment efficiency and stability of the coupled system.

### 3.6. Feasibility of agricultural irrigation applications

The treated greywater from the ICCW/MFC-SSF system consistently exceeded the requirements of the “Standards for Irrigation Water Quality” (GB 5084-2021). Key effluent parameters demonstrated high treatment efficiency: COD concentrations (10.5–30.6 mg L^−1^) were well below the standard limit (60–150 mg L^−1^), while suspended solids (16 mg L^−1^) and turbidity (3 NTU) complied with stringent thresholds. Microbial safety was notably achieved, with fecal coliform levels (0–360 MPN L^−1^) substantially lower than the permissible limit (10 000–40 000 MPN L^−1^). Effluent pH remained stable within 7.2–8.0, aligning with irrigation standards.

The comprehensive compliance of all critical parameters—particularly for organic and microbial control-confirms the suitability of the treated greywater for diverse agricultural applications, including paddy fields, dryland crops, and vegetable irrigation. Therefore, these findings establish a reliable basis for rural greywater recycling and present a sustainable approach to mitigating agricultural water scarcity.

## 4. Conclusion

The CW-MFC served as the core treatment unit, playing a pivotal role in system performance and pollutant removal. The SSF unit significantly enhanced nitrogen elimination through advanced polishing. DOM analysis *via* 3D-EEM-PARAFAC confirmed the effective degradation of labile, protein-like components, with the final effluent dominated by refractory fulvic-like substances. Microbial community analysis revealed distinct electroactive and metabolic niches, with Proteobacteria dominating the cathode and Firmicutes enriched at the anode, potentially underpinning the synergistic pollutant removal mechanisms. The system is best defined as an electrochemically enhanced constructed wetland, in which the embedded microbial fuel cell architecture (electrodes and circuit) primarily functions to establish an electrochemical microenvironment and stimulate relevant microbial functions, thereby improving treatment efficiency. Although laboratory studies have confirmed the bioelectricity generation potential of such iron–carbon-enhanced CW-MFCs, the success of this pilot-scale study lies fundamentally in the stable purification performance demonstrated during long-term field operation and the ecological mechanisms thereby revealed. The electrochemical enhancement mechanisms discussed (*e.g.*, micro-electrolysis, electron-shuttling effects) are inferred mainly from system design, microbial community data, and laboratory-scale electrochemical characterization, rather than from direct *in situ* electrochemical monitoring of the pilot-scale unit. It should be noted that a key methodological limitation of this field study is the absence of parallel control systems (*e.g.*, a conventional constructed wetland, an open-circuit CW-MFC, or an independent sand filtration unit), which precludes precise separation and quantification of the individual contributions from iron–carbon reactions, bioelectrochemical effects, and mere physical filtration. Under these circumstances, discussions concerning synergistic enhancement effects are based principally on the overall performance and internal diagnostic analysis of the integrated system, rather than on controlled comparative experiments. In summary, this study validates the long-term operational efficacy of the electrochemically enhanced wetland through pilot-scale field testing and provides an initial interpretation of its underlying microbial and electrochemical mechanisms, thereby laying a foundation for further mechanistic investigation and the design of controlled experiments.

However, the system exhibited a significant limitation in long-term phosphorus removal, with TP removal efficiency declining from an initial 87.5% to negligible levels. This decline was primarily due to adsorption saturation of the fillers and seasonal plant uptake. This underscores that while the ICCW/MFC-SSF system excels in organic and nitrogen removal, its phosphorus removal is non-sustainable under the current configuration.

Future work should therefore focus on integrating regenerative or continuous phosphorus removal strategies, such as the development of reusable adsorbent fillers or the incorporation of supplementary chemical precipitation, to ensure long-term operational stability and comprehensive nutrient management. Potential practical approaches could include the use of replaceable iron–carbon cartridges, the amendment of SSF media with Ca- or Mg-rich materials (*e.g.*, dolomite, calcite) to enhance precipitation, or the integration of a dedicated reactive filtration unit for phosphate capture. Furthermore, controlled laboratory-scale studies with the recommended control setups are essential to rigorously validate the proposed synergistic mechanisms and to generalize the findings across different operational contexts. This study provides both a viable technical framework for rural greywater reclamation and critical insights for the optimization of ecological treatment processes.

## Conflicts of interest

There are no conflicts to declare.

## Data Availability

All data generated or analysed during this study are included in this published article.
